# Eliglustat and cardiac comorbidities in Gaucher disease: a pharmacogenomic approach to safety and efficacy

**DOI:** 10.3389/fmed.2025.1535099

**Published:** 2025-03-17

**Authors:** Noor Ul Ain, Armaan Saith, Audrey Ruan, Ruhua Yang, Aaron Burton, Pramod K. Mistry

**Affiliations:** ^1^Department of Internal Medicine, Yale School of Medicine, New Haven, CT, United States; ^2^Specialty Pharmacy, Yale New Haven Hospital, New Haven, CT, United States; ^3^Department of Pediatrics, Yale School of Medicine, New Haven, CT, United States

**Keywords:** eliglustat, precision medicine, pharmacogenomcis, Gaucher disease, cardiac comorbidities, drug safety

## Abstract

**Introduction:**

Gaucher disease (GD), a lysosomal storage disorder, results from the accumulation of glycosphingolipids due to deficient lysosomal glucocerebrosidase activity. This pathological accumulation triggers immune activation, which paradoxically induces UDPglucose ceramide glucosyltransferase (UGCG), further exacerbating the metabolic defect. Eliglustat, a highly specific inhibitor of UGCG, functions as a substrate reduction therapy (SRT) and has demonstrated efficacy in reversing GD manifestations in clinical trials and real-world settings. Despite its established safety profile, preclinical studies have shown that supratherapeutic concentrations of eliglustat can inhibit ion channels involved in cardiac electrophysiology. However, pharmacogenomic-guided dosing ensures therapeutic efficacy while maintaining a wide safety margin, minimizing such risks. Nevertheless, lingering concerns regarding cardiac safety have persisted, particularly in patients with preexisting cardiac comorbidities.

**Methods:**

We report a single-center experience of eliglustat use in 13 patients with type 1 Gaucher disease (GD1) and concurrent cardiac comorbidities. Patients underwent standard cardiac evaluations, including electrocardiogram (EKG) with QTc interval assessment and echocardiogram. Eliglustat dosing was guided by CYP2D6 metabolizer status, and potential drug–drug interactions (DDIs) were carefully monitored.

**Results:**

Cardiac comorbidities included prior myocardial infarction (*n* = 2), aortic stenosis (*n* = 2), atrial fibrillation (*n* = 2), Wolff-Parkinson-White syndrome (*n* = 1), pericarditis (*n* = 1), premature ventricular complexes (*n* = 2), severe pulmonary arterial hypertension with right heart strain (*n* = 1), mitral annular calcification with diastolic dysfunction (*n* = 1), and mildly prolonged QTc interval (*n* = 1). No patients experienced arrhythmia, QTc prolongation, or arrhythmia-related symptoms. Treatment discontinuation was not required. All patients achieved expected therapeutic outcomes, as evidenced by serial reductions in glucosylsphingosine (GlcSph) levels and other disease indicators.

**Conclusion:**

This study represents the first real-world clinical evidence evaluating Eliglustat’s cardiac safety in a high-risk GD1 population. Unlike prior theoretical concerns derived from *in vitro* ion channel studies, our findings demonstrate that Eliglustat does not induce clinically significant cardiac events when administered according to pharmacogenomic guidelines. The misinformation regarding Eliglustat’s cardiotoxicity, largely driven by speculative interpretations rather than clinical data, is effectively countered by our findings, which show no significant QT prolongation or arrhythmias over a median treatment duration of 8 years.

## Introduction

Gaucher disease (GD) arises from biallelic mutations in the *GBA1* gene, resulting in defective acid *β*-glucosidase activity and the lysosomal accumulation of glucosylceramide (GlcCer) and glucosylsphingosine (GlcSph) (1, 2). The accumulation of these lipids triggers immune activation and paradoxically upregulates UDP-glucose ceramide glucosyltransferase (UGCG), further exacerbating the metabolic defect (3, 4). Accordingly, eliglustat, a selective UGCG inhibitor, has shown excellent efficacy in type 1 Gaucher disease (GD1) in both clinical trials and real-world settings (5–9). Pharmacogenomic dosing based on CYP2D6 genotype ensures therapeutic levels are achieved while maintaining a wide safety margin (10).

**Figure 1:**
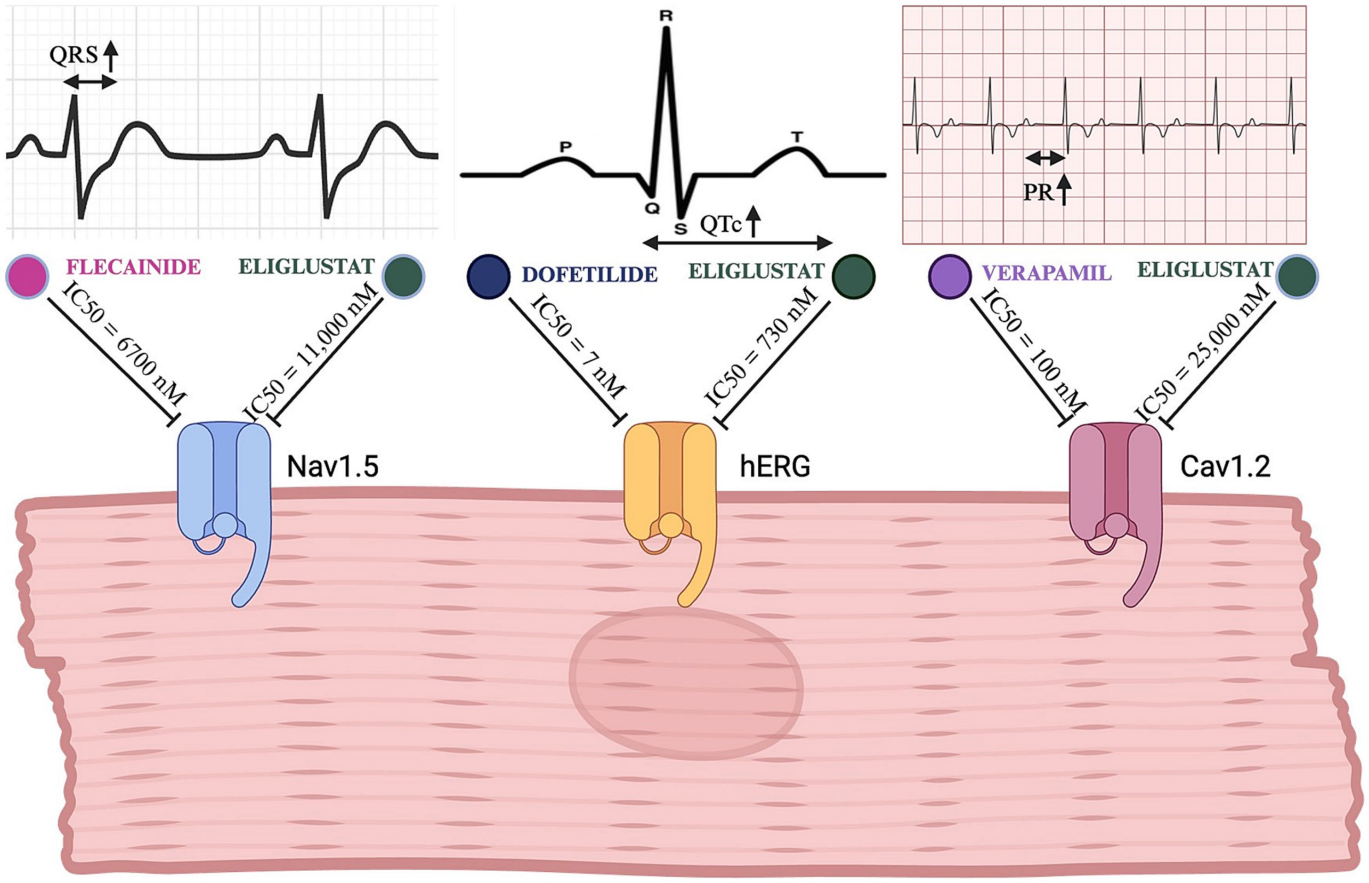
Comparative electrophysiological effects of eliglustat and clinically used channel-specific inhibitors. This figure illustrates the inhibitory effects of eliglustat on the Nav1.5, hERG, and Cav1.2 cardiac channels in comparison to specific channel inhibitors (flecainide, dofetilide, and verapamil, respectively). The top panels show EKG tracings highlighting the specific intervals/durations affected by each channel’s inhibition (QRS for Nav1.5, QT_c_ for hERG, and PR for Cav1.2). Created in BioRender. (23) BioRender.com/u76q381.

Preclinical studies of eliglustat demonstrated *in vitro* inhibition of several cardiac ion channels, including the human Ether-à-go-go Related Gene (hERG) channel (IC_50_ 730 nM), Nav1.5 (IC_50_ 11,000 nM), and Cav1.2 (IC_50_ 25,000 nM), as illustrated in Figure 1 (7). However, Clinical Evidence from real-world applications strongly suggests that pharmacogenomic-based dosing ensures plasma levels remain well below proarrhythmic thresholds. Despite this safety margin, concerns have persisted about potential cardiac risks, including arrhythmias such as polymorphic ventricular tachycardia, particularly in patients with preexisting cardiac conditions or long QT syndrome (11, 12). Notably, neither EMA nor FDA has revised cardiac safety labeling since regulatory approval, reflecting the absence of real-world safety concerns.

**Figure 2:**
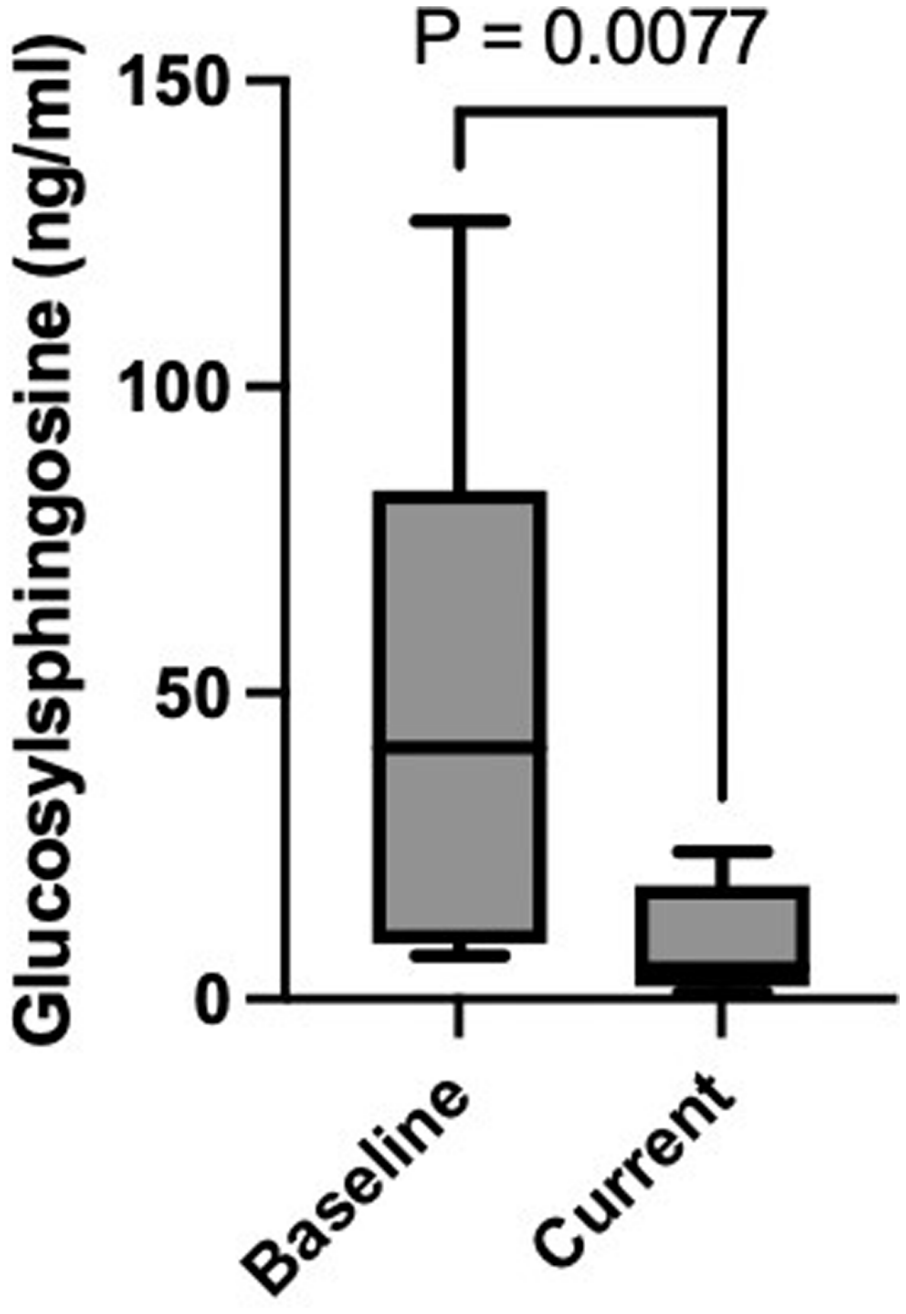
Baseline and current plasma GlcSph level. Figure highlights the statistically significant difference in plasma GlcSph level between baseline and after treatment with eliglustat SRT (*p* = 0.007).

To date, there remains a paucity of real-world clinical data evaluating the safety and efficacy of eliglustat substrate reduction therapy (SRT) in GD1 patients with significant cardiac comorbidities. To address this gap, we present a single-center experience involving 13 GD1 patients with concurrent cardiac conditions treated at a large tertiary referral center in the United States (Figure 2).

## Methods

### Study setting

The study was conducted at a tertiary referral center managing a large cohort of Gaucher disease (GD) patients. This specialized setting allowed for a robust evaluation of substrate reduction therapy (SRT) in a subset of GD1 patients with cardiac comorbidities.

### Study population

The study included 13 patients diagnosed with GD1 and concurrent cardiac comorbidities. GD diagnosis was confirmed by reduced leukocyte acid *β*-glucosidase activity and/or *GBA1* mutation analysis. Baseline characteristics and demographics are summarized in Table 1. Cardiac comorbidities included myocardial infarction (*n* = 2), atrial fibrillation (*n* = 2), aortic stenosis (*n* = 2), Wolff-Parkinson-White syndrome (*n* = 1), pericarditis (*n* = 1), severe pulmonary arterial hypertension with right bundle branch block (RBBB) (*n* = 1), premature ventricular contractions (PVCs) (*n* = 2), acquired long QT syndrome (*n* = 1), and diastolic dysfunction (*n* = 1). These patients were selected to evaluate eliglustat therapy outcomes in the context of cardiac comorbidities.

**Table 1:** Demographic characteristics of patients with cardiac comorbidities.

	*N* (%)
Gender	
Male	*n* = 6 (46)
Female	*n* = 7 (54)
Genotype	
p.Asn409Ser/p.Asn409Ser	*n* = 8 (61)
p.Asn409Ser/84GG	*n* = 2 (15)
p.Asn409Ser/IVS2 + 1	*n* = 1 (8)
p.Asn409Ser/p.Leu483Leu	*n* = 1 (8)
p.Asn409Ser/unidentified allele	*n* = 1 (8)
Cardiac comorbidities	
Aortic Stenosis	*n* = 2 (15)
Myocardial infarction	*n* = 2 (15)
Atrial fibrillation	*n* = 2 (15)
PVCs	*n* = 2 (15)
Pericarditis	*n* = 1 (8)
Diastolic dysfunction	*n* = 1 (8)
Mildly ↑ QTc interval	*n* = 1 (8)
WPW syndrome	*n* = 1 (8)
Severe PAH	*n* = 1 (8)
Duration of treatment, *N* (Range)	6.4 yrs. (1–13 years)

Table represents the demographic characteristics of our cohort of patients with underlying cardiac diseases. Quantitative data is expressed as percentages. PVCs, Premature ventricular contractions; WPW, Wolff Parkinsons White; PAH, Pulmonary Arterial Hypertension.

### Demonstrative case of pulmonary arterial hypertension, RBBB, LAFB, and SRT transition

This case exemplifies the significance of evaluating the safety and efficacy of eliglustat in patients with Gaucher disease and concurrent cardiac conditions. An adult patient, who underwent splenectomy for hypersplenism at age 14, developed severe pulmonary hypertension as a consequence of untreated Gaucher disease (13). Despite 20 years of intensive enzyme replacement therapy (ERT), which even required weekly dosing to achieve a modest response and significantly disrupted the patient’s life, residual disease persisted. The patient exhibited markedly elevated serum glucosylsphingosine (GlcSph) levels, >155-fold above the normal range, suggesting ongoing metabolic burden. Since the patient had achieved remission of visceral disease (normal liver volumes and liver function tests; the patient was asplenic), hematologic disease (normal blood counts and no marrow infiltration on MRI), and bone disease (stable bone density, no new avascular necrosis, and no active bone infiltration), the persistently elevated biomarker suggested a primary contribution from the lungs and pulmonary vascular disease. Studies of ERT targeting *in vivo* have shown that>95% of infused enzyme molecules are taken up the liver, spleen, and bone marrow; hence, pulmonary disease is a sanctuary site for Gaucher cells (14, 15).

**Figure 3:**
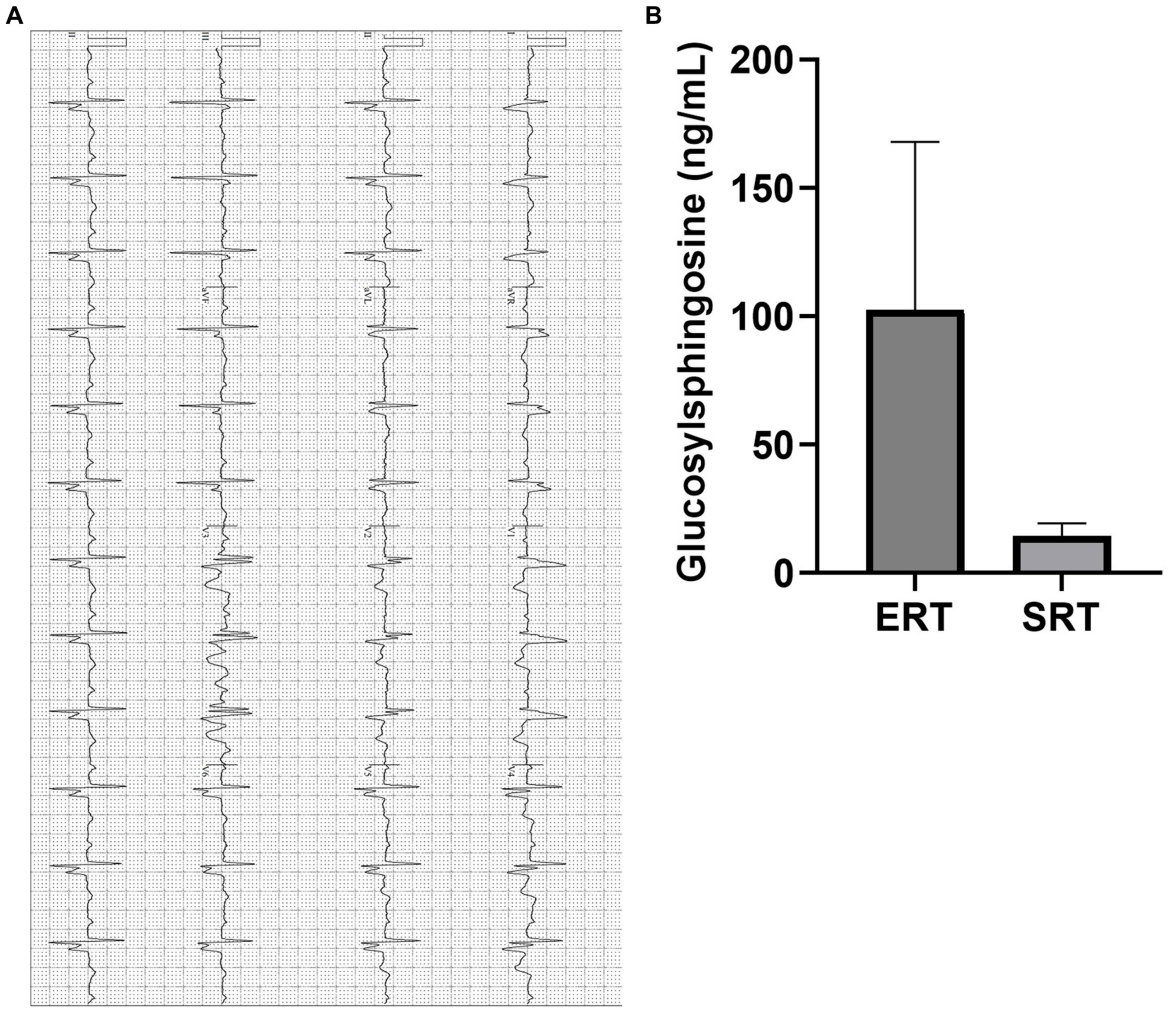
**(A)** EKG of a patient with PAH (Illustrative case). Electrocardiogram showing evidence of right bundle branch block (RBBB) with left axis bifascicular block (LAF) and mild atrial enlargement suggested by widening of the QRS complex, typical RSR’ pattern and left axis deviation. The QTc interval has remained within normal range on SRT, and symptomatically improved. PR: 214 ms, QTc: 460 ms, QRS: 159 ms. EKG, Electrocardiogram; PAH, Pulmonary Arterial Hypertension. **(B)** Plasma GlcSph levels on ERT and SRT of a patient with PAH (Illustrative case). Graph illustrates the reduction in plasma GlcSph level on more than 20 years of treatment with ERT vs. treatment with SRT for 1 year. There is 91% reduction in plasma GlcSph level. Error bars represent SD. GlcSph, Glucosylsphingosine, ERT, Enzyme replacement therapy; SRT, Substrate reduction therapy; PAH, Pulmonary Arterial Hypertension; SD, Standard Deviation.

Cardiac evaluation revealed right bundle branch block (RBBB) and left anterior fascicular block (LAFB) on electrocardiogram (EKG). Given the patient’s CYP2D6 metabolizer status, eliglustat was introduced as an alternative therapy. The transition to eliglustat led to marked clinical improvement, with the patient reporting significant reductions in dyspnea, fatigue, and palpitations. Follow-up assessments demonstrated stabilization of cardiac conduction intervals on EKG and an impressive 91% reduction in GlcSph levels, from 155 ng/mL to 14 ng/mL after just 1 year of SRT (Figures 3A,B). These findings reinforce the potential therapeutic advantage of eliglustat in managing Gaucher disease patients with pulmonary and cardiac involvement, particularly in cases where ERT may be suboptimal.

### Baseline evaluations

Comprehensive baseline evaluations included complete blood count (CBC), comprehensive metabolic profile (CMP), GD-specific biomarkers (chitotriosidase and GlcSph), ferritin, iron saturation, lipid profile, immunofixation electrophoresis, EKG (QTc interval, QRS, and PR intervals, arrhythmias), and echocardiography to assess cardiac function. Standard imaging testing, MRI of the abdomen lower extremities, and DXA scans were also performed.

### Drug dosing

Pharmacogenomics-guided dosing of eliglustat was implemented based on each patient’s CYP2D6 metabolizer status, determined via genotyping.

DNA analysis for CYP2D6 genotyping is conducted using allele-specific real-time polymerase chain reaction (RT-PCR) to identify single-nucleotide polymorphisms (SNPs), insertions or deletions (indels), hybrid alleles, hybrid tandem variants, and copy number variants (CNVs) within the CYP2D6 gene. This assay enables the detection and classification of variant CYP2D6 alleles, including *2, *3, *4, *5 (deletion), *6, *7, *8, *9, *11, *12, *13 (hybrid), *13, *15, *17, *29, *31, *35, *36 (hybrid), *36 + *10 (hybrid tandem), *40, *41, *42, *49, *53, *59, and *68 (hybrid), as well as gene duplications (DUPs) with copy number (CN) designation. The *1 allele represents the reference (wild-type) sequence detected at the assessed loci (16).

Patients were classified as poor (PM), intermediate (IM), extensive (EM), or ultra-rapid metabolizers (UM). Poor metabolizers, who have significantly reduced CYP2D6 enzyme function, received 84 mg once daily to minimize drug accumulation. Intermediate metabolizers, with moderately reduced enzyme activity, were prescribed 84 mg twice daily, while extensive metabolizers, who exhibit normal CYP2D6 activity, followed the standard recommended dose of 84 mg twice daily.

### Drug–drug interactions

In addition to pharmacogenomic stratification, potential drug–drug interactions (DDIs) were assessed. Medications that inhibit CYP2D6 (e.g., fluoxetine, paroxetine) or CYP3A4 (e.g., ketoconazole) were reviewed, and additional monitoring was implemented where necessary. We adhered to the recommended procedures for patients on concurrent QT-prolonging medications, conducting serial electrocardiographic assessments to ensure cardiac safety (Supplementary Table 1). In our study, no drug–drug interactions (DDIs) necessitated dose adjustments for any patient.

### Outcome measures

Primary outcomes assessed safety and efficacy of eliglustat in GD1 patients with cardiac comorbidities. Hematological responses, visceral organ volumes, and GD-specific biomarkers (GlcSph and chitotriosidase) were measured. Serial EKGs monitored QTc prolongation, QRS and PR interval changes, and arrhythmias. Concomitant medications were reviewed to mitigate drug–drug interaction risks.

### Disease-specific parameters

Efficacy was evaluated by monitoring GD-specific biomarkers (GlcSph and chitotriosidase) alongside hematological and visceral parameters. Biochemical responses and their impact on disease progression were tracked.

Plasma GlcSph was measured using LC–MS/MS: For sample preparation, 25 pg. of internal standard (N,N-Dimethylsphingosine; Matreya, Pleasant Gap, PA) was added to a 2.0 mL Eppendorf tube and evaporated to dryness under nitrogen gas. Plasma samples (20 μL) were mixed with 1,000 μL of chloroform:methanol (2:3, v/v), vortexed for 5 min, and centrifuged at 16,000 × g for 3 min. The supernatant was transferred to a fresh tube, followed by the addition of 200 μL chloroform and 520 μL water for liquid–liquid extraction. The solution was vortexed for 3 min, centrifuged at 16,000 × g for 3 min, and the lower organic phase was collected into an autosampler vial. The aqueous phase was re-extracted with chloroform, and the pooled lower phases were combined in the autosampler vial. The sample was then evaporated under nitrogen gas and reconstituted in 100 μL methanol:water (9:1, v/v) for liquid chromatography–tandem mass spectrometry (LC–MS/MS) analysis. A calibration curve was generated using lyso-GL1 standards (Matreya, Pleasant Gap, PA) processed identically to the plasma samples.

Reconstituted samples were analyzed using an LC–MS/MS system consisting of a Waters Acquity UPLC coupled to an API-5000 triple-quadrupole mass spectrometer. Separation of lyso-GL1 (glucosylsphingosine, lyso-Gb1) and other matrix components was achieved on an Acquity BEH C18 column (2.1 × 50 mm, 1.7 μm) under gradient elution conditions, with mobile phase A comprising 0.1% formic acid in water and mobile phase B consisting of 0.1% formic acid in acetonitrile. Mass spectrometry was performed in selected ion monitoring (SIM) mode with the following transitions: m/z 462.5 → 282.4 for lyso-GL1 and m/z 490.3 → 292.4 for N,N-Dimethylsphingosine (17).

### Statistical analysis

Statistical analyses were conducted using R Studio and IBM SPSS Statistics. Paired *t*-tests assessed normally distributed variables (GlcSph, QTc, PR intervals) as determined by the Shapiro–Wilk test. Non-normally distributed variables (QRS intervals) were analyzed using the Wilcoxon rank-sum test. Missing data were excluded from the analysis.

### Ethical considerations

Informed consent was obtained from all participants, allowing data collection from their standard care management for this longitudinal study.

## Results

### Patient characteristics

Baseline characteristics and demographics are summarized in Table 1. Among the 13 patients with GD1 and cardiac comorbidities, 8 (61%) were homozygous for the p.Asn409Ser/p.Asn409Ser mutation. The remaining 5 patients (39%) were heteroallelic, with the following genotypes: p.Asn409Ser and 84GinsertionG (*n* = 2; 15%), IVS2 + 1 (*n* = 1; 8%), p.Leu483Leu (*n* = 1; 8%), and a complex allele (*n* = 1).

### Duration of therapy

The median duration of eliglustat therapy was 8 years (range: 1–13 years). Eleven patients (85%) remained on therapy throughout the study. One patient discontinued therapy due to dry skin and scaling, a known adverse effect of eliglustat. Another patient died at the age of 87 following complications from Parkinson’s disease and aspiration pneumonia.

### Cardiac response

Despite a wide spectrum of pre-existing cardiac comorbidities, no new cardiac events were observed during the study. Specific responses included:

- A patient with recurrent pericarditis for over a decade requiring innumerable hospital admissions despite pericardiectomy achieved complete remission on eliglustat.
- A patient with severe pulmonary arterial hypertension (PAH) and right heart strain showed significant clinical improvement after transitioning from enzyme replacement therapy (ERT) to eliglustat monotherapy. This improvement correlated with a marked reduction in GlcSph levels.
- One patient was newly diagnosed with Wolff-Parkinson-White (WPW) syndrome during routine EKG monitoring and underwent successful accessory bundle ablation. Post-procedure, the patient tolerated eliglustat without complications.

No significant changes were observed in EKG parameters (PR, QRS, QTc intervals) during the study period. Furthermore, no patient experienced syncope, palpitations, or Torsade de Pointes on follow-up EKGs.

### Biomarker response

Patients transitioning from ERT to eliglustat demonstrated a marked reduction in plasma GlcSph levels. The mean plasma GlcSph level decreased from 50.8 ng/mL pre-treatment to 9.5 ng/mL (normal <1.0 ng/mL), representing an 81% reduction (*p* = 0.007) (Table 2).

**Table 2:** Hematological, visceral, and EKG interval values at baseline and post treatment with eliglustat.

Patient	Current age (Years)	Age category (Years)	Sex	*GBA* genotype	Leucocyte acid *β*-glucosidase activity (nmol/h/mg)	CYPD26 status	Cardiac Comorbidities	Age category at diagnosis of GD (Years)	Duration of treatment (Years)	Eliglustat dosage
1	70	>60	F	p.Asn409Ser/84GG	NA	IM	Myocardial Infarction	<18	3	84 mg BiD
2	56	40–60	F	p.Asn409Ser/p.Asn409Ser	0.2	PM	Aortic Stenosis	18–39	1	84 mg OD
3	60	40–60	F	p.Asn409Ser/p.Asn409Ser	0.19	IM	Myocardial Infarction	18–39	13	84 mg BiD
4	63	>60	M	p.Asn409Ser/p.Asn409Ser	NA	EM	Pericarditis	40–60	4	84 mg OD*
5	59	40–60	M	p.Asn409Ser/p.Asn409Ser	NA	EM	Atrial Fibrillation	40–60	9	84 mg BiD
6	86	>60	M	p.Asn409Ser/p.Asn409Ser	NA	PM	Diastolic Dysfunction	18–39	9	84 mg OD
7	60	40–60	F	p.Asn409Ser/p.Asn409Ser	NA	IM	Mildly ↑ QTc interval	40–60	4	84 mg BiD
8	74	>60	F	p.Asn409Ser/?	NA	PM	PVCs	>60	8	84 mg BiD
9	14	<18	F	p.Asn409Ser/p.Asn409Ser	0.3	IM	WPW syndrome	<18	2	42 mg BiD**
10	55	>60	F	p.Asn409Ser/L444P	NA	PM	PVCs	18–39	12	84 mg OD
11	63	>60	M	p.Asn409Ser/84GG	NA	EM	Aortic Stenosis	<18	9	84 mg BiD
12	73	>60	M	p.Asn409Ser/p.Asn409Ser	NA	EM	Atrial Fibrillation	18–39	9	84 mg OD***
13	68	>60	M	p.Asn409Ser/IVS2 + 1	NA	PM	PAH/RBBB /LAFB	<18	1	84 mg OD

Patient	Hemoglobin (gm/dl)	Platelet (x1000/ul)	Liver volume	Spleen volume	GlcSph (ng/ml)		QTc interval (ms)		PR interval (ms)		QRS interval (ms)	
			MN	MN	Baseline	Current	Baseline	Current	Baseline	Current	Baseline	Current
1	9.5	365	NA	Splenectomized	41	8.4	395	394	172	148	78	82
2	13.1	110	1.01	6.59	28.3	21.2	417	NA	166	NA	86	NA
3	13.3	184	0.92	1.53	NA	5	406	414	141	155	90	88
4	15	140	0.71	2.25	83	14	428	348	164	164	102	108
5	15.1	188	0.77	2.04	9	5	430	416	178	186	94	84
6	15.2	197	0.89	3.36	99.3	2	481	524	176	212	150	168
7	13.1	251	0.59	0.88	9	1.6	422	425	142	159	87	82
8	12.5	279	0.76	Splenectomized	NA	15	NA	NA	NA	NA	NA	NA
9	13.4	256	NA	7.96	70	4	408	NA	80	NA	76	NA
10	14.3	104	1.12	3.8	7	1	NA	431	NA	172	NA	98
11	17.8	142	1.11	4.76	46.4	24.06	413	459	201	221	82	135
12	12.5	91	0.61	1.53	127.1	4	397	406	150	164	184	89
13	16	251	0.68	Splenectomized	39	18.5	NA	468	NA	228	NA	162

Table lists the actual values for hematological, visceral, disease biomarker and EKG intervals. The patients were divided into the following age categories: <18, 18–39, 40–60, >60 years. The difference in EKG intervals pre and post treatment was statistically and clinically insignificant. All patients showed expected response to eliglustat SRT with significant decline in GlcSph. GlcSph, Glucosylsphingosine; MN, Multiples of normal organ size; NA, Not available; PAH, Pulmonary arterial hypertension; WPW, Wolff Parkinson White syndrome; PVC, Premature ventricular contractions; PM, Poor metabolizer; IM, Intermediate metabolizer; EM, Extensive metabolizer; BID, twice daily; OD, Once daily. Leucocyte acid β-glucosidase activity normal range: 7.5–14.5 nmol/h/mg protein. Plasma glucosylsphingosine normal range: ≤1 ng/mL. *Dose reduced due to adverse effect of diarrhea. **Dose adjusted according to weight (<50 kg). ***Dose adjusted due to persistent atrial fibrillation and uncontrolled hypertension.

### Adverse effects

Eliglustat was well tolerated across the cohort. Importantly, no significant EKG changes, including QTc interval prolongation, were observed. No syncopal episodes or adverse cardiac events occurred during the treatment period, supporting the favorable long-term safety profile of eliglustat in patients with cardiac comorbidities.

## Discussion

This study provides valuable real-world evidence on the safety and efficacy of eliglustat as a substrate reduction therapy (SRT) in patients with type 1 Gaucher disease (GD1) and concurrent cardiac comorbidities. By implementing pharmacogenomic-guided dosing based on CYP2D6 metabolizer status, our findings demonstrate that eliglustat can be safely administered in this high-risk population without significant adverse cardiac events. This is particularly noteworthy given previous concerns regarding eliglustat’s potential to induce cardiac arrhythmias, primarily based on *in vitro* studies showing inhibition of key ion channels, including hERG, Nav1.5, and Cav1.2 (7). In our cohort, there were no significant changes in QTc, PR, or QRS durations, and no incidences of torsades de pointes, atrioventricular (AV) block, or life-threatening arrhythmias following the initiation of eliglustat therapy. These results align with existing clinical trial data and pharmacokinetic/pharmacodynamic (PK/PD) modeling. A Phase I study by Ruskin et al. (7) found no clinically significant electrocardiographic (EKG) changes at a mean maximum plasma concentration (Cmax) of 236.8 ng/mL. Furthermore, PK/PD modeling predicts that even at an eliglustat Cmax of 500 ng/mL—a level only achievable with concomitant CYP3A4 and CYP2D6 inhibitors—EKG intervals would increase only modestly (QTc by 13 ms, QRS by 7 ms, and PR by 22 ms). However, pharmacogenomic-guided dosing ensures that actual Cmax levels remain significantly lower, with typical values of 12.1–25 ng/mL in extensive metabolizers, 44.6 ng/mL in intermediate metabolizers, and 75 ng/mL in poor metabolizers—all well below the threshold associated with clinically meaningful EKG changes (7).

From a regulatory perspective, both the EMA and FDA have maintained Eliglustat’s cardiovascular safety labeling without revisions, indicating that no significant safety concerns have emerged from post-marketing surveillance or adverse event reporting. This further supports the conclusion that Eliglustat does not present an increased cardiac risk when dosed appropriately. The use of eliglustat in patients with pre-existing cardiac conditions has not been extensively evaluated in clinical trials. However, a pooled analysis of four completed eliglustat trials identified only two cases of serious cardiac arrhythmias—one involving ventricular tachycardia and another atrioventricular (AV) block, which necessitated withdrawal from the trial in one patient. Notably, the AV block was successfully managed with dose adjustment, indicating that appropriate dosing strategies can mitigate such risks. Additionally, 2.8% of participants reported palpitations as an adverse event, though these were transient, self-limiting, and did not require treatment discontinuation (18).

Only a single case of severe bradycardia and hemodynamic compromise has been reported in the literature, attributed to an overdose of 94 capsules of eliglustat—a dose far exceeding the therapeutic range (19). This underscores that, when administered within recommended guidelines, eliglustat maintains a strong cardiac safety profile. Furthermore, careful monitoring for potential drug–drug interactions (DDIs) remains an essential component of safe prescribing, ensuring optimal patient outcomes even in those with underlying cardiac disease. While *in vitro* studies have demonstrated eliglustat’s inhibitory effects on hERG, Nav1.5, and Cav1.2 channels, these findings must be interpreted in the context of clinically relevant concentrations. Eliglustat’s IC50 values for these channels (hERG: 730 nM; Nav1.5: 11,000 nM; Cav1.2: 25,000 nM) are orders of magnitude higher than those of clinically used channel specific inhibitors like dofetilide (hERG: 7 nM), verapamil (Cav1.2: 100 nM), and flecainide (Nav1.5: 6,700 nM) (20). This suggests that the concentrations required to significantly affect cardiac electrophysiology far exceed those achieved in clinical use.

Moreover, the simultaneous inhibition of multiple ion channels by eliglustat may exert a protective electrophysiological effect rather than a proarrhythmic one. For instance, the combined inhibition of calcium (Cav1.2) and potassium (hERG) currents may create a balancing effect that stabilizes the cardiac action potential plateau, reducing the likelihood of arrhythmias (7). Similarly, inhibition of the late Nav1.5 current could help prevent cellular calcium overload and early afterdepolarizations, both of which are key triggers for torsades de pointes (21). These mechanistic insights reinforce that eliglustat, when used at therapeutic doses, is unlikely to pose a clinically significant proarrhythmic risk.

All patients in our cohort exhibited significant reductions in glucosylsphingosine (GlcSph) levels, accompanied by clinical stabilization or improvement. The notable clinical benefits observed in patients with complex cardiac conditions, including the remission of recurrent pericarditis and improvement in right heart strain associated with severe pulmonary arterial hypertension (PAH), highlight eliglustat’s efficacy in managing GD1 patients with cardiac comorbidities. Certain cardiac complications represent rare but direct manifestations of GD, and their poor response to enzyme replacement therapy (ERT) underscores the limitations of ERT due to inefficient enzyme delivery beyond visceral, hematologic, and skeletal compartments (13, 22). These findings align with previous studies demonstrating eliglustat’s effectiveness in reducing GlcSph levels and improving hematological and visceral parameters in GD1 patients. Furthermore, our results expand the therapeutic scope of eliglustat, showing greater efficacy compared to ERT monotherapy in managing disease burden within sanctuary sites such as the lungs and pericardium, where ERT penetration is limited (14).

## Conclusion

Our findings suggest that eliglustat SRT, when dosed according to pharmacogenomic profiling and monitored for drug–drug interactions (DDIs), is both safe and effective in GD1 patients with cardiac comorbidities. This study highlights the critical role of personalized medicine in optimizing outcomes for GD patients with significant cardiac risks. Future prospective studies with larger cohorts are needed to confirm these findings and explore the long-term impact of eliglustat on cardiac health in this population.

## Limitations

While this study offers valuable insights, certain limitations must be acknowledged. The small sample size and single-center design restrict the generalizability of the findings to broader GD1 populations. Additionally, the retrospective nature of the study introduces the potential for selection and recall bias, and the absence of a control group limits direct comparative analyses. Despite these constraints, the long follow-up duration (mean 6.4 years) and consistent cardiac monitoring strengthen the reliability and clinical relevance of our observations.

## Data Availability

The original contributions presented in the study are included in the article/Supplementary material, further inquiries can be directed to the corresponding author.
