# Supplementary material for: Eliglustat and cardiac comorbidities in Gaucher disease: a pharmacogenomic approach to safety and efficacy
Source: Front Med (Lausanne). 2025 Mar 17;12:1535099. doi: 10.3389/fmed.2025.1535099 (PMC11956841; doi:10.3389/fmed.2025.1535099)
Supplement: Supplementary file 1 [file Table_1.docx]

**Supplementary Table 1. Eliglustat Drug-Drug Interactions**

| **Drug Class** | **Examples** | **Clinical Effect** | **Recommendation** |
| --- | --- | --- | --- |
| CYP2D6 Inhibitors | Paroxetine, Fluoxetine, Bupropion, Quinidine | Increases Eliglustat levels → Risk of toxicity | Avoid in intermediate/extensive metabolizers |
| CYP3A4 Inhibitors | Ketoconazole, Itraconazole, Clarithromycin, Ritonavir | Increases Eliglustat levels → Avoid in poor metabolizers | Avoid in poor metabolizers |
| CYP3A4 Inducers | Rifampin, Carbamazepine, Phenytoin, St. John’s Wort | Reduces Eliglustat levels → Loss of efficacy | Avoid co-administration |
| QT-Prolonging Drugs | Amiodarone, Sotalol, Moxifloxacin, Citalopram | QT prolongation → Risk of arrhythmia | Use with caution; avoid if high arrhythmia risk |
